# Structure analysis of precursor alloy and diffusion during dealloying of Ag–Al alloy†

**DOI:** 10.1039/c7ra12915g

**Published:** 2018-03-05

**Authors:** Runwei Zhang, Xu Wang, Zhichao Zhang, Jacob C. Huang, Feng Shi, Ming Wu

**Affiliations:** School of Mechanical Engineering, Liaoning Shihua University Fushun 113001 P. R. China wangxu@lnpu.edu.cn wx1979875@hotmail.com; Institute for Advanced Study, Department of Materials Science & Engineering, City University of Hong Kong Kowloon Hong Kong

## Abstract

Nanoporous silver (NPS) was fabricated by dealloying Ag–Al alloy ribbons with nominal compositions of 30, 35 and 40 at% Ag (corresponding to hypoeutectic composition, eutectic composition and hypereutectic composition, respectively). The microstructures of the Ag–Al precursor and as-dealloyed samples were observed using a scanning electron microscope (SEM) and a transmission electron microscope (TEM) as well as *via* focused ion beam (FIB) technique. We concluded that with the increase in Ag content from 30 to 40 at%, the diameter of ligament increased from 70 ± 15 nm to 115 ± 35 nm. Due to the method of crystalline solidification and the distribution of α-Al(Ag) and γ-Ag_2_Al phases, the as-dealloyed Ag_35_Al_65_ alloy exhibited a homogeneous ligament/pore structure, whereas the microstructures of Ag_30_Al_70_ and Ag_40_Al_60_ showed thinner and coarser ligament structures, respectively.

## Abstract 2

Nanoporous silver (NPS) was fabricated by dealloying Ag–Al alloy ribbons with nominal compositions of 30, 35 and 40 at% Ag (corresponding to hypoeutectic composition, eutectic composition and hypereutectic composition, respectively).

## Graphical abstract

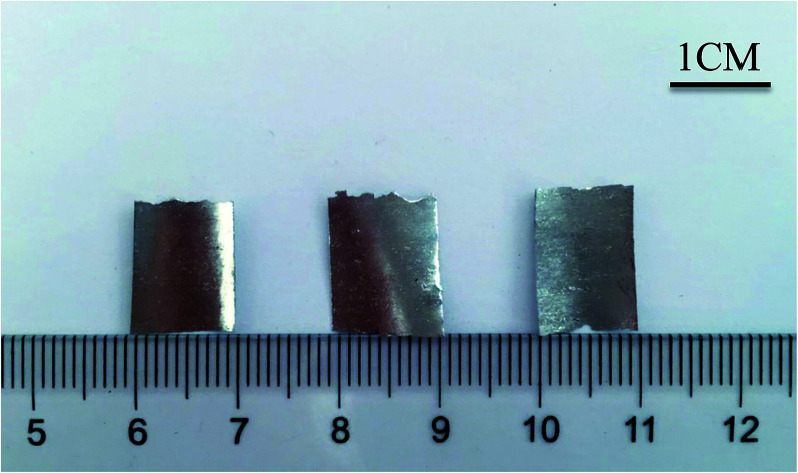

## Introduction

Nanoporous metals have recently attracted considerable attention in many applications such as catalysis, optics, sensors, actuators and energy storage because of their special structures and properties.^1–4^ In addition, nanoporous metals exhibit a three-dimensional (3D), bi-continuous ligament/pore structures. Recently, nanoporous metals have been commonly fabricated by template and dealloying methods, which include the replication of porous templates and the removal of one or more active metal elements from an alloy, respectively.^5–10^

Compared with the template method, the dealloying method is more flexible in modulating the microstructure as well as the size of the ligament/pore structure. Furthermore, the dealloying processes and microstructures of nanoporous metals are affected by many factors such as precursor alloy composition and dealloying solution,^11^ temperature^12^ and time.^13^ Therefore, investigating the dealloying process and the formation mechanism of nanoporous metals is essential.

In recent years, an increasing number of studies have been conducted to explore the fabrication of nanoporous metals by dealloying. Correspondingly, many nanoporous metals have been fabricated through dealloying from binary or ternary alloy systems, such as Cu–Pt,^14^ Ag–Au,^15^ Cu–Au^16^ and Au–Ag–Pt,^17^ most of those binary or ternary alloy systems possess single-phase solid solubility across all compositions. However, studies on the fabrication of nanoporous metals have not merely concentrated on the single-phase alloy systems; bi-phase (even multi-phase) alloys or compounds, such as Ag–Al,^18^ Al–Cu,^19^ Mg–Cu^20^ and Al–Ag–Au,^21^ have also been proved to be appropriate for the fabrication of nanoporous metals or composites. Consequently, the mechanical behaviour and the formation mechanism of nanoporous composites and metals during the dealloying process are worthy of investigation. For example, nanoporous Cu is prepared *via* chemical dealloying of Ag–Cu alloys and the mechanical behaviour of bulk nanoporous Cu is discussed.^22^ The microstructure and phase evolution during dealloying of the bi-phase Ag–Al alloy are reported; the results suggest that the dealloying of α-Al(Ag) and Ag_2_Al starts simultaneously, and subsequently, the residual Ag_2_Al is dealloyed.^23^

Owing to their economical costs, relatively high strengths and high electrical and thermal conductivities,^24,25^ Ag, Al and Cu have been used as substitutes for Au. In addition, some dealloying strategies for the preparation of nanoporous Ag dealloyed from Ag–Al^26^ and Ag–Cu alloys have been reported.^27,28^ However, the dealloying behaviour and microstructure evolution of the initial Ag alloys still needs to be explained in detail. Compared with the single-phase Au–Ag binary alloy system, binary alloy systems with more than one phase, such as Ag–Al and Ag–Cu alloys, complicate the dealloying process and microstructural evolution. Therefore, further research on dealloying Ag, Al and Cu is needed.

In addition, the effects of the alloy composition on microstructures (especially the ligament/pore size) of the resulting nanoporous structures were investigated.^29,30^ However, detailed studies on the effect of phase formation and distribution on the microstructure of nanoporous silver (NPS) are relatively few. Therefore, in this study, we designed and prepared Ag–Al precursor alloys with different compositions based on Ag–Al phase diagrams. From the Ag–Al phase diagram,^31^ the eutectic point was found to be 67 at% for Al at 840 K (567 °C). Thus, the Ag contents in Ag_30_Al_70_, Ag_35_Al_65_ and Ag_40_Al_60_ corresponded to hypoeutectic, eutectic, and hypereutectic compositions, respectively. Moreover, we briefly discussed the phase distribution involving halo formation during the solidification process of alloys. Halo formation means that one phase is formed around another phase that was formed initially.^32^ This process commonly occurs during the solidification of off-eutectic alloys.

## Materials and methods

**Fig. 1:**
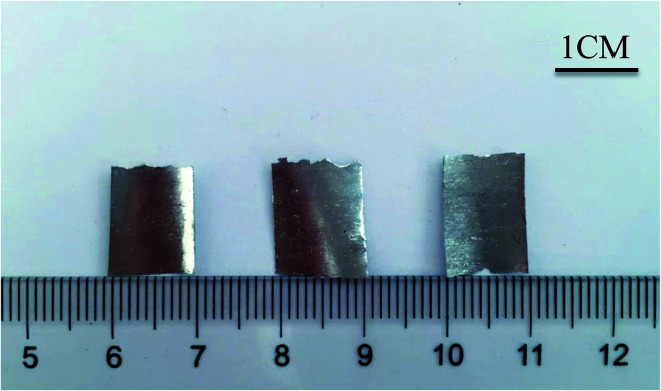
Photograph of the Ag–Al alloy ribbons.

The Ag–Al precursor alloys with Ag compositions of 30, 35 and 40 at% were prepared by melting pure metals (Al and Ag in 99.9 wt% purity) in a high-frequency induction melting furnace. The alloy was heated to a temperature that was 100 °C above the melting point of the alloy and was kept for 10–20 minutes. The Ag–Al alloy ingot was then re-melted and was rapidly solidified into ribbons (thickness: 40–60 μm; width: ∼10 mm) *via* single-roller melt-spinning with copper mould water cooling plant (cooling rate: 50–300 °C s^−1^; rotational speed: 2500 revolutions per minute). The Ag–Al alloy samples are depicted in Fig. 1. The melt-spun Ag–Al ribbons were dealloyed by immersing them in 200 ml, 5 wt% dilute hydrochloric acid (HCl) at 60 °C (333 K). To observe the dealloying behaviour of the Ag–Al alloy during the dealloying process, Ag–Al ribbons were etched for 0, 5, 10, 20 and 30 min. The dealloyed specimens were sequentially rinsed several times with distilled water and ethyl alcohol.

The phase patterns of precursor alloys and as-dealloyed samples were collected using an X-ray diffractometer (XRD) with a scanning speed set at 2° min^−1^. 2*θ* scans were performed between 20° and 90°. The microstructures of the Ag–Al precursor and the as-dealloyed samples were observed using a Hitachi SU8000 scanning electron microscope (SEM) and an FEI Tecnai G2 F20 transmission electron microscope (TEM) *via* a focused ion beam (FIB) apparatus (Seiko SMI3050). The ligament sizes of nanoporous Ag in SEM images were measured by a Java-based public image processing software imageJ. The content of Al and Ag in the precursor and the as-dealloyed samples was detected through an energy dispersive X-ray spectroscopy (EDS) analyser attached to SEM.

## Results and discussion

### Characterization of melt-spun Ag–Al precursor alloy

**Fig. 2:**
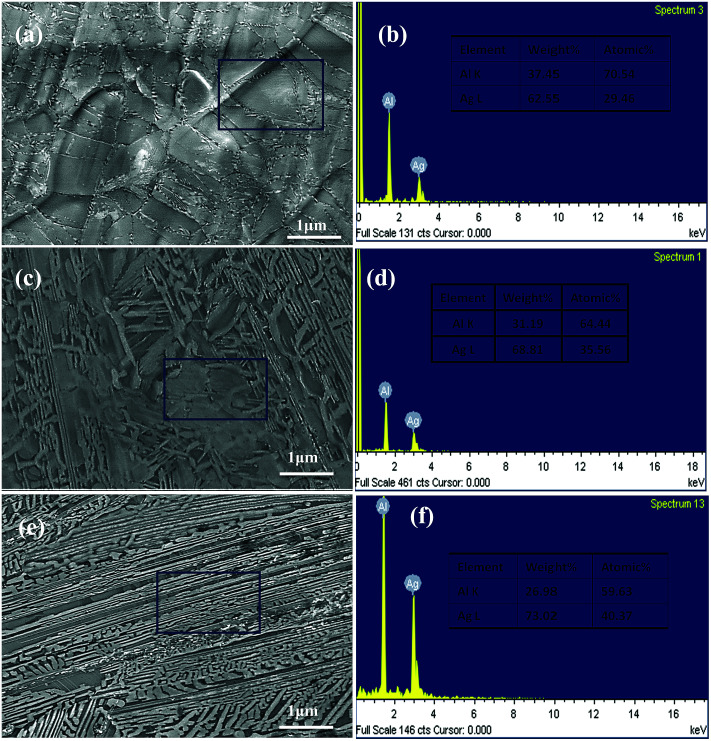
SEM micrographs of the (a) Ag_30_Al_70_, (c) Ag_35_Al_65_ and (e) Ag_40_Al_60_ melt-spun samples (plan view); (b), (d) and (f) EDS results of Ag_30_Al_70_, Ag_35_Al_65_ and Ag_40_Al_60_ precursor alloy samples.

**Fig. 3:**
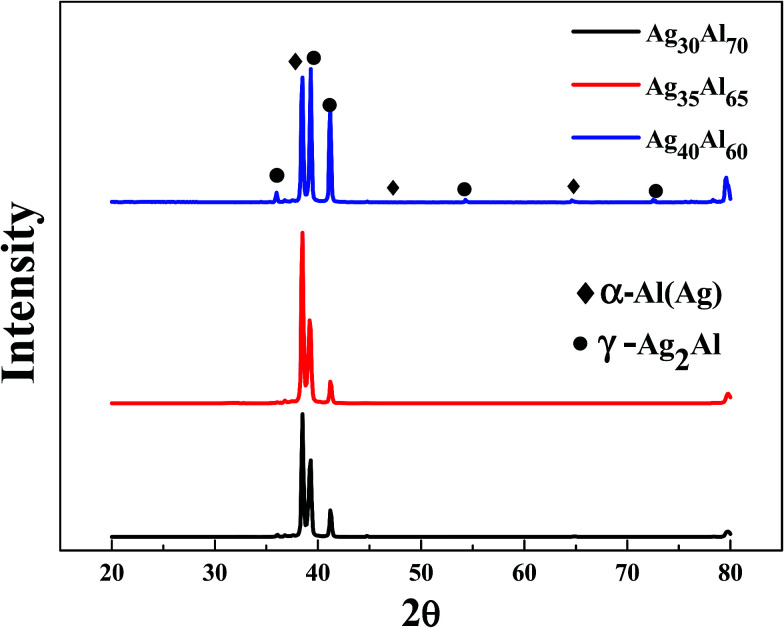
XRD patterns of the melt-spun Ag–Al ribbons.

The SEM micrographs (plan view) of the Ag_30_Al_70_, Ag_35_Al_65_ and Ag_40_Al_60_ melt-spun samples are shown in Fig. 2, which also shows that distinct grain boundaries (or phase boundaries) appeared in the Ag_30_Al_70_ and Ag_35_Al_65_ as-spun alloys, and the morphology of the Ag_35_Al_65_ precursor alloy was more uniform than that of the Ag_30_Al_70_ alloy. The corresponding EDS results of the Ag–Al precursor alloys are shown in Fig. 2(b), (d) and (f). The results were consistent with our designation of the alloy composition in the initial Ag–Al alloys. Fig. 3 shows the XRD patterns of the melt-spun Ag–Al ribbons with an Ag content of 30–40 at%. The XRD patterns depicted two phases in the melt-spun Ag–Al alloys, namely, the phases of the α-Al(Ag) solid solution and γ-Ag_2_Al intermetallic compound.

### Microstructure of as-dealloyed Ag–Al ribbons

**Fig. 4:**
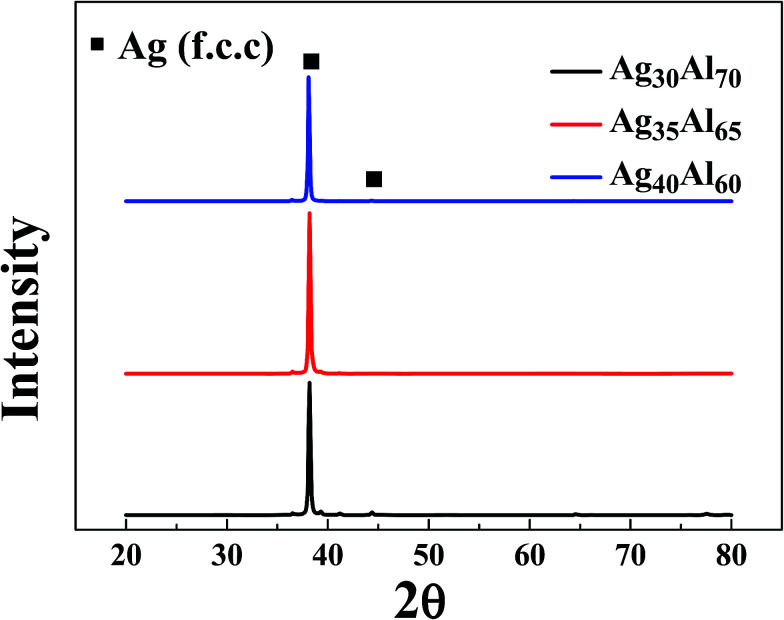
XRD patterns of Ag–Al samples under the dealloyed condition for 30 min.

Fig. 4 shows the XRD patterns of the as-dealloyed Ag–Al ribbons. According to the XRD results, the diffraction peaks corresponding to α-Al(Ag) and γ-Ag_2_Al disappeared. In addition, the as-dealloyed ribbons with Ag content ranging from 30 to 40 at% in the precursor alloys contained only one phase: the single face-centered-cubic (FCC) Ag phase. In addition, the α-Al(Ag) and Ag_2_Al phases were completely dealloyed, whereas Al from these two phases leached out entirely.

**Fig. 5:**
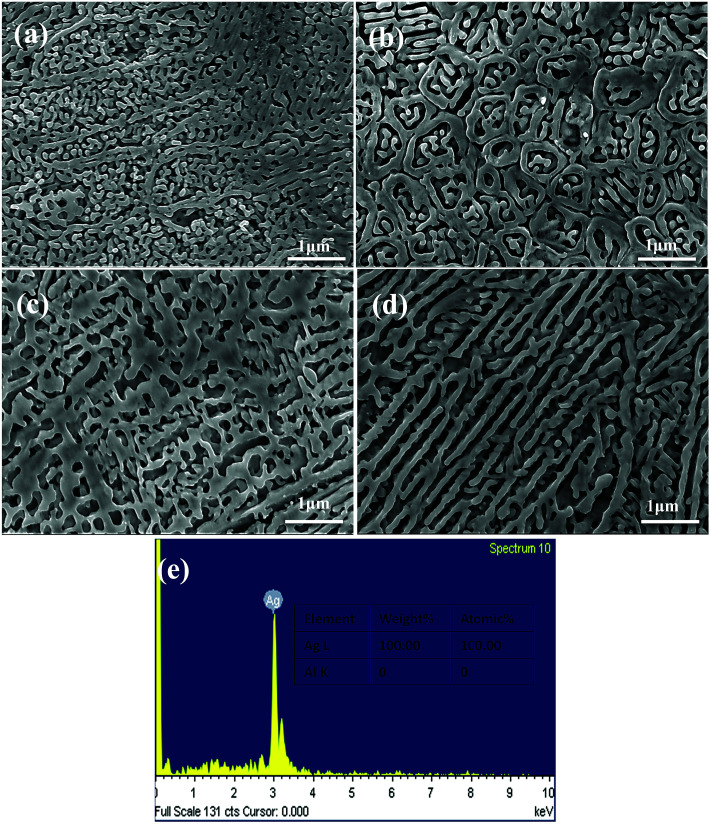
SEM micrographs of the Ag_30_Al_70_ samples (plan view) under the dealloyed conditions for different periods of time: (a) 5 min, (b) 10 min, (c) 20 min, and (d) 30 min; (e) EDS pattern of Ag_30_Al_70_ sample dealloyed for 30 min.

Fig. 5 shows the microstructures of the NPS ribbons (plan view) dealloyed from the Ag_30_Al_70_ alloys for different durations in a 5 wt% HCl solution at 60 °C. The morphology of the resulting NPS ribbon dealloyed for 30 min exhibited an inhomogeneous, ligament-channel structure. Moreover, some deep channels (or large cracks) were formed at the early stage of the dealloying process, and they became more uniform as time progressed. After being dealloyed for 30 min, the diameter of the ligament became coarse and changed from 60 ± 15 to 72 ± 35 nm. Fig. 5(e) shows the EDS pattern of the Ag_30_Al_70_ alloy dealloyed for 30 min. This indicated that there was no Al element that could be detected in the as-dealloyed Ag_30_Al_70_ sample.

**Fig. 6:**
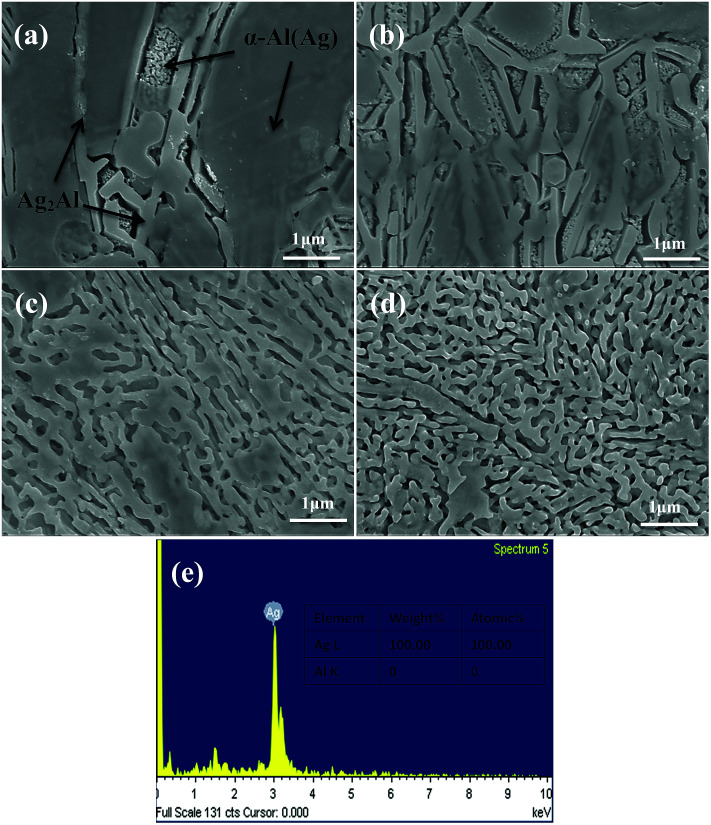
SEM micrographs of the Ag_35_Al_65_ samples (plan view) under the dealloyed conditions for different periods of time: (a) 5 min, (b) 10 min, (c) 20 min, and (d) 30 min; (e) EDS pattern of Ag_35_Al_65_ sample dealloyed for 30 min.

**Fig. 7:**
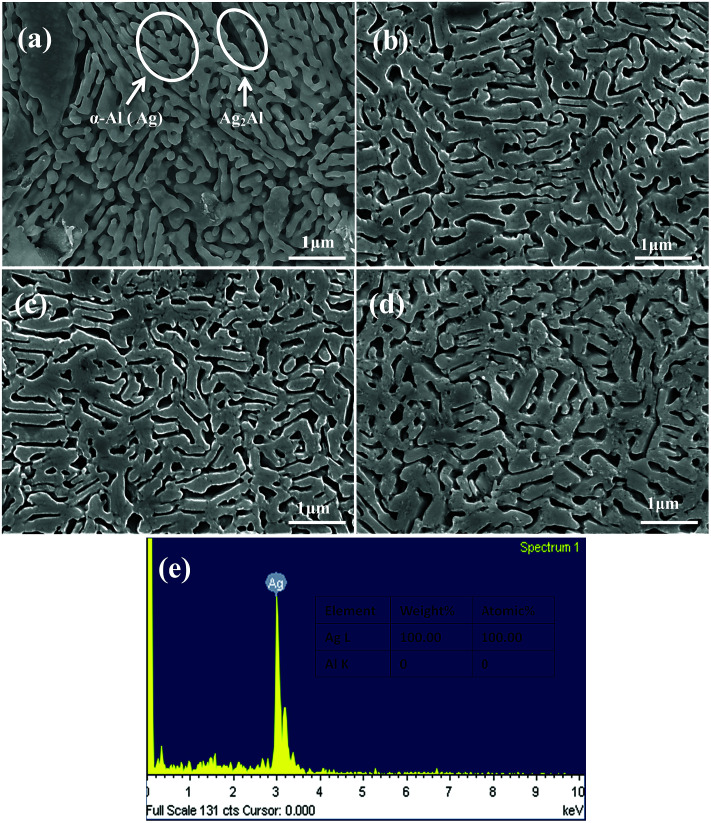
SEM micrographs of the Ag_40_Al_60_ samples (plan view) under the dealloyed conditions for different periods of time: (a) 5 min, (b) 10 min, (c) 20 min, and (d) 30 min; (e) EDS pattern of Ag_40_Al_60_ sample dealloyed for 30 min.

The microstructures of the NPS ribbons (plan view) dealloyed from the Ag_35_Al_65_ alloys for different durations are shown in Fig. 6. It was obvious that the final porous structure of the resulting NPS ribbon (Fig. 6d) was more homogeneous than those of Ag_30_Al_70_ (Fig. 5d) and Ag_40_Al_60_ (Fig. 7d) alloy ribbons, and it exhibited a bi-continuous, interpenetrating ligament/pore structure without the appearance of deep channels (or cracks). The phase distribution is shown in Fig. 6(a). After being dealloyed for 30 min, the diameter of ligament became coarse from 40 ± 15 to 90 ± 35 nm. Fig. 6(e) shows the EDS pattern of Ag_35_Al_65_ alloy dealloyed for 30 min. This indicated that there was no Al element that could be detected in the as-dealloyed Ag_35_Al_65_ sample.

Fig. 7 shows the morphology of the NPS ribbons (plan view) dealloyed from the Ag_40_Al_70_ alloys and a nearly homogeneous interpenetrating ligament-channel structure. The phase distribution is shown in Fig. 7(a). However, the diameter of the ligament dealloyed for 30 min from the Ag_40_Al_70_ alloy reached approximately 150 nm and was much larger than those of the Ag_30_Al and Ag_35_Al alloys. This result suggested that the size of the ligament became larger with the increase in Ag content. After being dealloyed for 30 min, the diameter of the ligament coarsened from 90 ± 15 to 115 ± 35 nm. Fig. 7(e) shows the EDS pattern of Ag_40_Al_60_ alloy dealloyed for 30 min. This indicated that no Al element could be detected in the as-dealloyed Ag_40_Al_60_ sample.

From the EDS patterns (Fig. 5e, 6e and 7e) of Ag_30_Al_70_, Ag_35_Al_65_ and Ag_40_Al_60_ alloys, respectively, we concluded that after dealloying, there was no residual Al element in the Ag–Al alloy samples. Thus, the EDS patterns were consistent with the XRD results (Fig. 4). However, it is unreasonable to say that there was absolutely no Al left. Moreover, because the amount of residual Al was too small, it could be ignored.^18^

### Diffusion of Ag atoms during the dealloying process

**Fig. 8:**
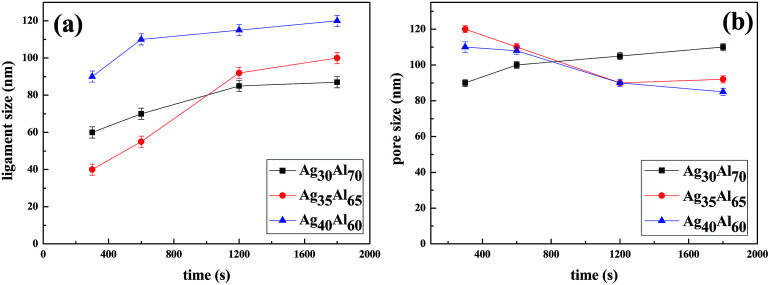
Statistics of ligament size (a) and pore size (b) with the increasing dealloying time in different alloy samples.

The micro-scale changes in ligaments and pores during the dealloying process are shown in Fig. 8. To clearly understand the formation mechanism of the nanoporous structure, the dependence of the growth kinetics of ligament on the diffusion of Ag was investigated. The analysis of the statistical data suggested that the coarsening of the ligament and the pores followed a nonlinear relationship, and the ligament sizes increased with an increase in Ag contents. Moreover, consistent with the observations in the SEM results, the ligament size of the Ag_35_Al_65_ alloy was found to be dimensionally similar to the nanopore size, whereas there were large differences between the ligament and nanopore sizes in Ag_30_Al_70_ and Ag_40_Al_60_.

As reported, the surface diffusion, *D*_s_, which is the main factor of the coarsening mechanism, can be estimated by the following equation:1
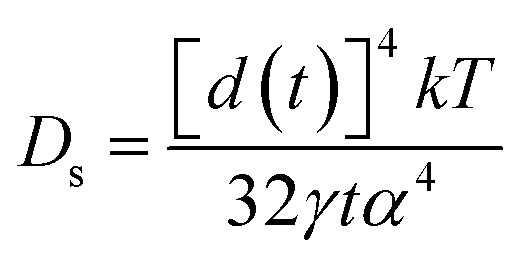
here, *d* is the diameter of the ligament at time *t*, *γ* is the surface energy of Ag (1.24 J m^−2^),^33^*α* is the lattice parameter of Ag (0.4088 nm), *D*_s_ is the coefficient of surface diffusion of Ag, *k* is the Boltzmann constant (1.380648 × 10^−23^ J K^−1^) and *T* is the dealloying temperature. Thus, the surface diffusivity results of Ag_30_Al_70_, Ag_35_Al_65_ and Ag_40_Al_60_ were estimated to be 6.2 × 10^−17^ m^2^ s^−1^, 1.5 × 10^−16^ m^2^ s^−1^ and 4.0 × 10^−16^ m^2^ s^−1^, respectively. Therefore, slight changes in the alloy composition significantly affected the diffusivity of Ag, and the results from this equation explained the coarsening behaviour of the Au nanostructure during dealloying.^34^

**Fig. 9:**
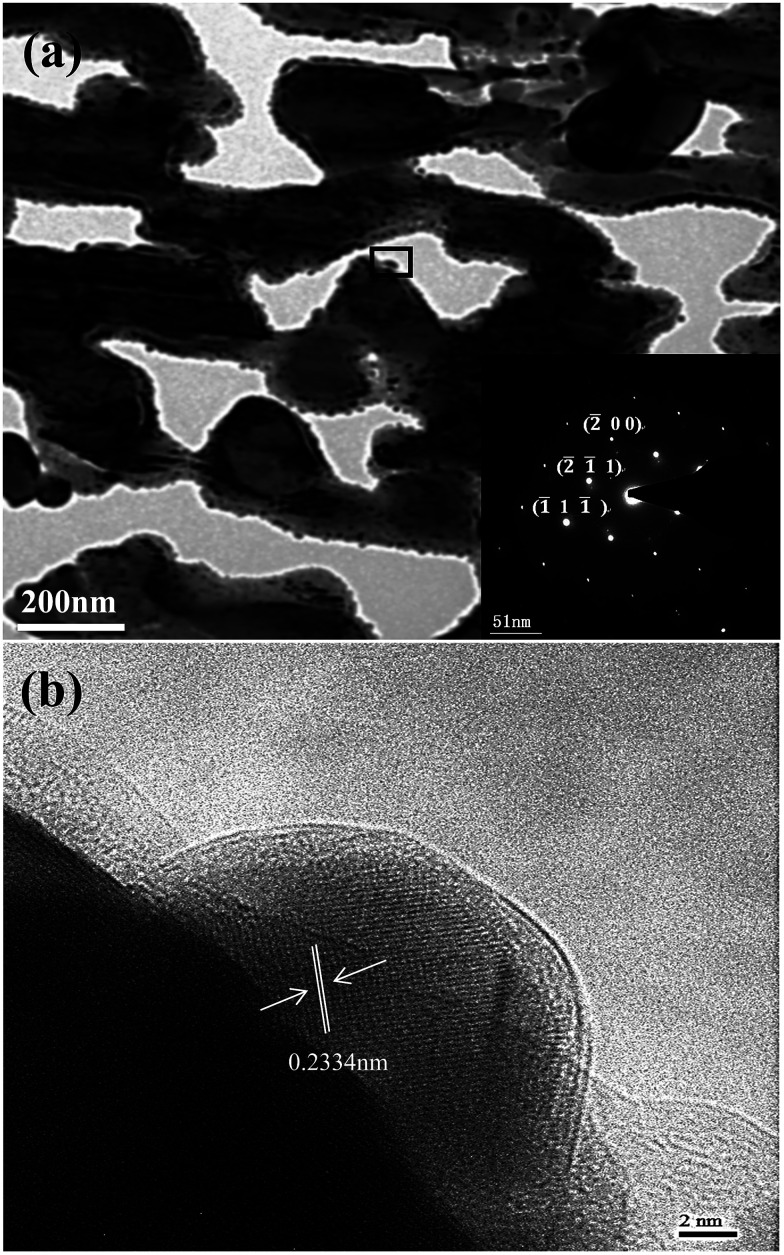
(a) TEM bright-field image with SAED pattern inset for Ag_35_Al_65_ ribbons dealloyed for 30 min; (b) selected areas (marked by rectangles in (a)) magnified HRTEM bright-field image.

Fig. 9 shows the bright field TEM image with the inset corresponding to the elected-area diffraction (SEAD) pattern of the Ag_35_Al_65_ alloy. Fig. 9(a) revealed that the ligament comprised polycrystalline Ag nanoparticles. The distinct morphology exhibited a characteristic ligament/pore structure and was consistent with the SEM observation (Fig. 6). The selected area electron diffraction (SEAD) pattern comprised polycrystalline rings, corresponding to the (2̄ 1̄ 1), (2̄ 0 0) and (1̄ 1 1̄) planes of FCC Ag. The high-resolution transmission electron microscope (HRTEM) image (Fig. 9b) was taken from Fig. 9(a) and marked by a rectangle. The lattice fringe of the ligament could be clearly observed in the HRTEM image (Fig. 9b), and the inter-planar distance was estimated to be 0.2334 nm, which was in good agreement with that of the {111} planes of Ag, further confirming the fact that the nanoparticle was FCC Ag. Apparently, there were certain interface orientation relationships between the ligaments and the Ag atoms (Fig. 9b).

### Ligament coarsening and corrosion mechanism

**Fig. 10:**
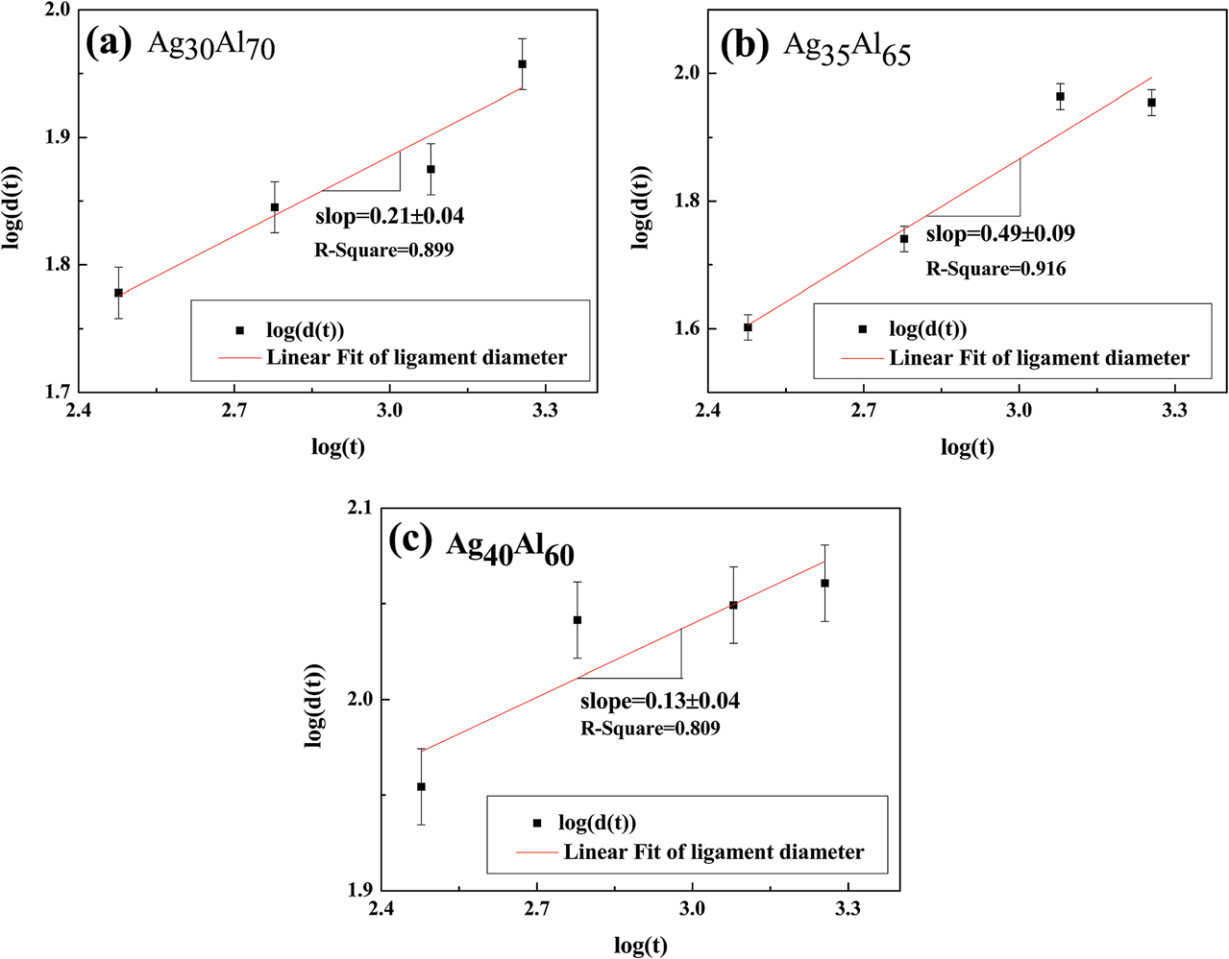
Log–log plot of ligament diameter *versus* dealloying time for samples.

Fig. 10 shows the mean diameter of the ligament as a function of time, and the fitting shows a general linear relationship. Thus, the following relationship could be predicted between the diameter of the ligament (*d*) and dealloying time (*t*):^35^

2 *d*^*n*^ = *KtD*_s_

here, *K* is a constant, *D*_s_ is the surface diffusivity at a certain temperature and *n* is the coarsening exponent of the ligaments. By logarithmic transformation, the equation could be expressed as follows:

3 *n* ln *d* = ln(*KD*_s_) + ln *t*

Eqn (3) shows a relationship between *d* and *t*, *i.e.*, *d* ∝ *t*^1/*n*^. Furthermore, the slope of the fitting linear curve is equal to 1/*n*. However, there is not a definite relationship between the diameter of ligament (*d*) and dealloying time (*t*). The time dependence of the ligament coarsening is susceptible to many factors; for example, the ligament diameter exhibits a time dependence *d* ∝ *t*^0.13^ ≈ *t*^1/8^ when dealloying^36^ and *d* ∝ *t*^1/5^ when sintering.^37^ The slope of the fitting linear curve (shown in Fig. 10) obtained from the Ag_35_Al_65_ (*d* ∝ *t*^0.49±0.04^) alloy is steeper than those obtained from the Ag_30_Al_70_ (*d* ∝ *t*^0.21±0.09^) and Ag_40_Al_60_ (*d* ∝ *t*^0.13±0.04^) alloys, suggesting that the rate of ligament coarsening of Ag_35_Al_65_ is faster than those of the other two alloys.

The slopes (0.21 ± 0.04, 0.49 ± 0.09 and 0.13 ± 0.04) of the fitting lines for different compositions implied a variable coarsening mechanism at different conditions. Therefore, it is reasonable to assume that Ag content and the phase distribution in the precursor alloy were possible reasons for such results.

Referring to the binary Ag–Al diagram,^31^ we discovered that prior to the formation of the α-Al(Ag)/γ-Ag_2_Al eutectic structure, the primary α-Al(Ag) phase first precipitated during rapid solidification of the Ag_30_Al_70_ and Ag_35_Al_65_ alloys, whereas the primary γ-Ag_2_Al phase precipitated in preference to the α-Al(Ag) phase during rapid solidification of the Ag_40_Al_60_ alloy. Therefore, large differences in the microstructures and morphologies of Ag_30_Al_70_, Ag_35_Al_65_ and Ag_40_Al_60_ initial alloys appeared.^19,23^ The observation from Fig. 2(a), (c) and (d) confirmed this prediction. Thus, these differences were mainly determined by the formation and distribution of the two phases [α-Al(Ag) and γ-Ag_2_Al]. By comparing the microstructures of Ag_30_Al_70_, Ag_35_Al_65_ and Ag_40_Al_60_ during the dealloying process shown in Fig. 5–7, we found that the morphology of initial alloys and distribution of α-Al(Ag) and γ-Ag_2_Al could influence the nanoporous structures significantly. However, there were some differences in the alloy solidification and nanoporous structure between our report and a former research.^18^ The reason may be that the experimental conditions and dealloying parameters presented in our research were different from those presented in the former research.

According to a new model of halo formation in directional solidification proposed by Nave,^32^ for Ag_40_Al_60_, the nucleation and growth of the eutectic structure directly occurred from the primary phase γ-Ag_2_Al. However, for the Ag_30_Al_70_ and Ag_35_Al_65_ alloys, when the temperature rapidly decreased (the undercooling for nucleation was significant), a halo phase γ-Ag_2_Al was generated from the primary phase α-Al(Ag), and it developed around the α-Al(Ag) phase before the eutectic formation occurred. Therefore, the α-Al(Ag) phase was enveloped by the γ-Ag_2_Al phase (Fig. 6a). There would be more grain boundaries (or phase boundaries) in this kind of alloy so that the intergranular corrosion would be facilitated, and this distinct phase distribution could improve the dealloying ability.

**Fig. 11:**
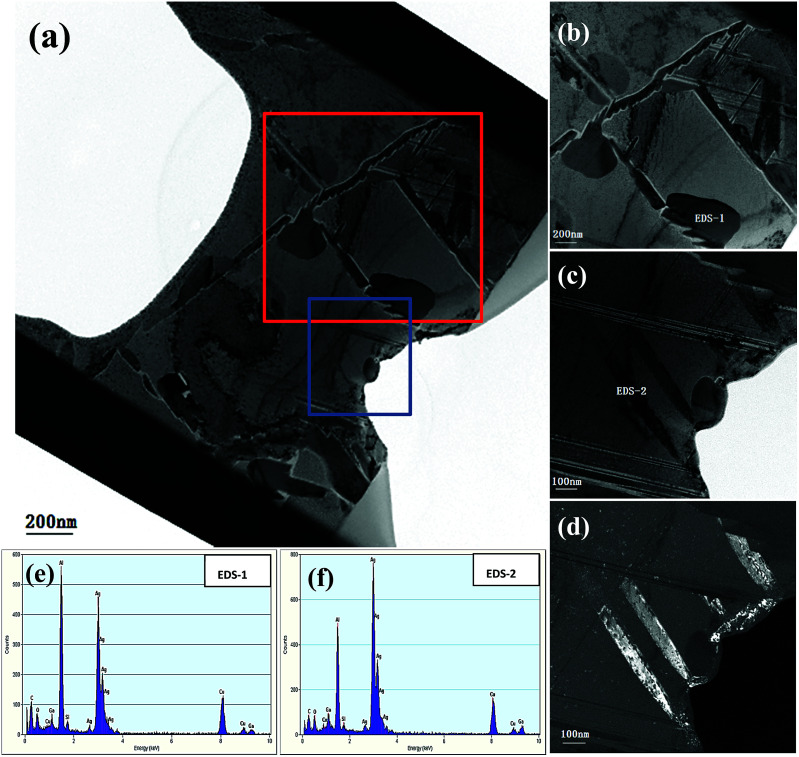
(a–c) HRTEM images of a cross section incised *via* FIB and corresponding DF-TEM (d) and EDS (e, f) results of the Ag_35_Al_65_ initial alloy.

Fig. 11(a) shows the HRTEM image of a whole cross section incised *via* FIB from the initial Ag_35_Al_75_ alloy sample and reveals a clear morphology of the internal structure. Fig. 11(b) and (c) depict the HRTEM photographs of the areas selected from Fig. 11(a), marked by red and blue rectangles, respectively. We can clearly the observe grain boundaries distributed in the alloy interior from Fig. 11(a). Fig. 11(d) is the corresponding dark field TEM (DF-TEM) image of Fig. 11(c). There are two spots (EDS-1 and EDS-2) located in Fig. 11(b) and (c) which are detected by EDX, and the EDS results are shown in Fig. 11(e) and (f), respectively. According to the EDS patterns, the bright and dark regions, corresponding to Ag_2_Al and α-Al(Ag), respectively, show a distinct distribution of different phases.

As can be observed from Fig. 5 and 6, the intergranular corrosion corresponding to the etching at the grain boundary (phase boundary) occurred at the initial stage of the dealloying process for the Ag_30_Al_70_ and Ag_35_Al_65_ alloys, resulting in the appearance of deep channels (or cracks) at the phase boundary. Since the phase boundary or grain boundary was less stable than the grains inside, the preferential etching of Al occurred from the phase boundaries. Thus, as is shown in Fig. 6(a), the microstructure dealloyed from the α-Al(Ag) phase exhibited a 3D bi-continuous ligament/pore structure. Additionally, the structure dealloyed from γ-Ag_2_Al was a pore-wall structure. This phenomenon was beneficial for the dissolution of Al and for increasing the dealloying rate, due to which the dealloying rate of Ag_40_Al_60_ became relatively slow. However, compared with the more active phase α-Al(Ag), which could be easily dealloyed in the electrolyte, the inert phase γ-Ag_2_Al was less prone to directly react with the HCl solution. As a result, the ligaments of as-dealloyed Ag_40_Al_60_ were seriously coarsened (Fig. 7d). In addition, due to the synthesised effect of α-Al(Ag) and γ-Ag_2_Al phases, Ag_35_Al_65_ (eutectic composition) exhibited a homogeneous ligament/pore structure, whereas the microstructures of Ag_30_Al_70_ (hypoeutectic composition) and Ag_40_Al_60_ (hypereutectic composition) exhibited inhomogeneous thinning and coarsening of ligaments, respectively. This result confirmed that the synergetic dealloying of α-Al(Ag) and γ-Ag_2_Al in the two-phase Ag–Al alloys was conducive to the formation of NPS with a homogeneous porous structure.^19^

## Conclusions

(1) In summary, the composition of precursor alloys significantly affected the dealloying process and the formation of NPS. The size of the ligament in the as-dealloyed samples became larger with the increase in Ag content in the precursor alloy.

(2) Due to the variation of Ag content, the morphology in each precursor alloy was different. For the Ag_30_Al_70_ and Ag_35_Al_65_ alloys, when the temperature rapidly decreased (the undercooling for nucleation was significant), a halo phase γ-Ag_2_Al was generated from the primary phase α-Al(Ag), and it developed around the α-Al(Ag) phase before the eutectic formation occurred. In addition, for the Ag_40_Al_60_ alloy, the nucleation and growth of the eutectic structure directly occurred from the primary phase γ-Ag_2_Al with no halos.

(3) The formation of the halo phase accelerated the ligament coarsening rate because it provided more phase boundaries. With the synthesised effect of α-Al(Ag) phase and γ-Ag_2_Al phase, Ag_35_Al_65_ (eutectic composition) exhibited a homogeneous ligament/pore structure, whereas the microstructures of Ag_30_Al_70_ (hypoeutectic composition) and Ag_40_Al_60_ (hypereutectic composition) with thinning and coarsening ligaments, respectively, exhibited inhomogeneous structures.

## Conflicts of interest

There are no conflicts to declare.

## Supplementary Material

RA-008-C7RA12915G-s001
