# Supplementary material for: Structure analysis of precursor alloy and diffusion during dealloying of Ag–Al alloy
Source: RSC Adv. 2018 Mar 5;8(17):9462–70. doi: 10.1039/c7ra12915g (PMC9078667; doi:10.1039/c7ra12915g)
Supplement: RA-008-C7RA12915G-s001 [file RA-008-C7RA12915G-s001.pdf]

Electronic Supplementary Material (ESI) for RSC Advances.
This journal is © The Royal Society of Chemistry 2018

# Figures

Fig.1 Photograph of the Ag-Al alloy ribbons

Fig.2 SEM micrographs of the (a) $Ag_{30}Al_{70}$, (c) $Ag_{35}Al_{65}$ and (e) $Ag_{40}Al_{60}$ melt-spun samples (plan view); (b), (d) and (f) EDS results of $Ag_{30}Al_{70}$, $Ag_{35}Al_{65}$ and $Ag_{40}Al_{60}$ precursor alloy samples.

Fig.3 XRD patterns of the melt-spun Ag-Al ribbons

Fig.4. XRD patterns of Ag-Al samples under the dealloyed condition for 30 min.

Fig.5 SEM micrographs of the $Ag_{30}Al_{70}$ samples (plan view) under the dealloyed conditions for different periods of time: (a) 5 min, (b) 10 min, (c) 20 min, and (d) 30 min; (e) EDS pattern of $Ag_{30}Al_{70}$ sample dealloyed for 30 min.

Fig.6 SEM micrographs of the $Ag_{35}Al_{65}$ samples (plan view) under the dealloyed conditions for different periods of time: (a) 5 min, (b) 10 min, (c) 20 min, and (d) 30 min; (e) EDS pattern of $Ag_{35}Al_{65}$ sample dealloyed for 30 min.

Fig.7 SEM micrographs of the $Ag_{40}Al_{60}$ samples (plan view) under the dealloyed conditions for different periods of time: (a) 5 min, (b) 10 min, (c) 20 min, and (d) 30 min; (e) EDS pattern of $Ag_{40}Al_{60}$ sample dealloyed for 30 min.

Fig.8 Statistics of ligament size (a) and pore size (b) with the increasing dealloying time in different alloy samples.

Fig.9(a) TEM bright-field image with SAED pattern inset for $Ag_{35}Al_{65}$ ribbons dealloyed for 30 min; (b) Selected areas (marked by rectangles in Fig.9(a) magnified HRTEM bright-field image.

Fig.10 Log-log plot of ligament diameter versus dealloying time for samples.

Fig.11(a-c) HRTEM images of a cross section incised via FIB and corresponding DF-TEM (d) and EDS (e,f) results of the $Ag_{35}Al_{65}$ initial alloy.

`

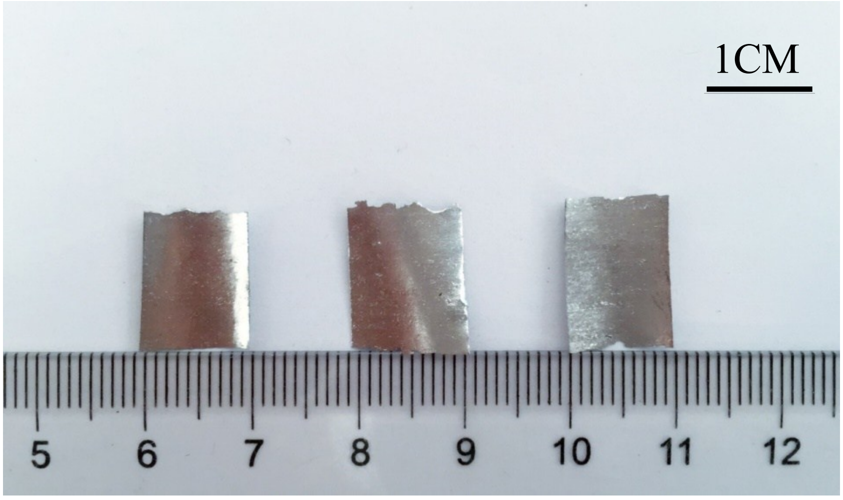


**FIG. 1**

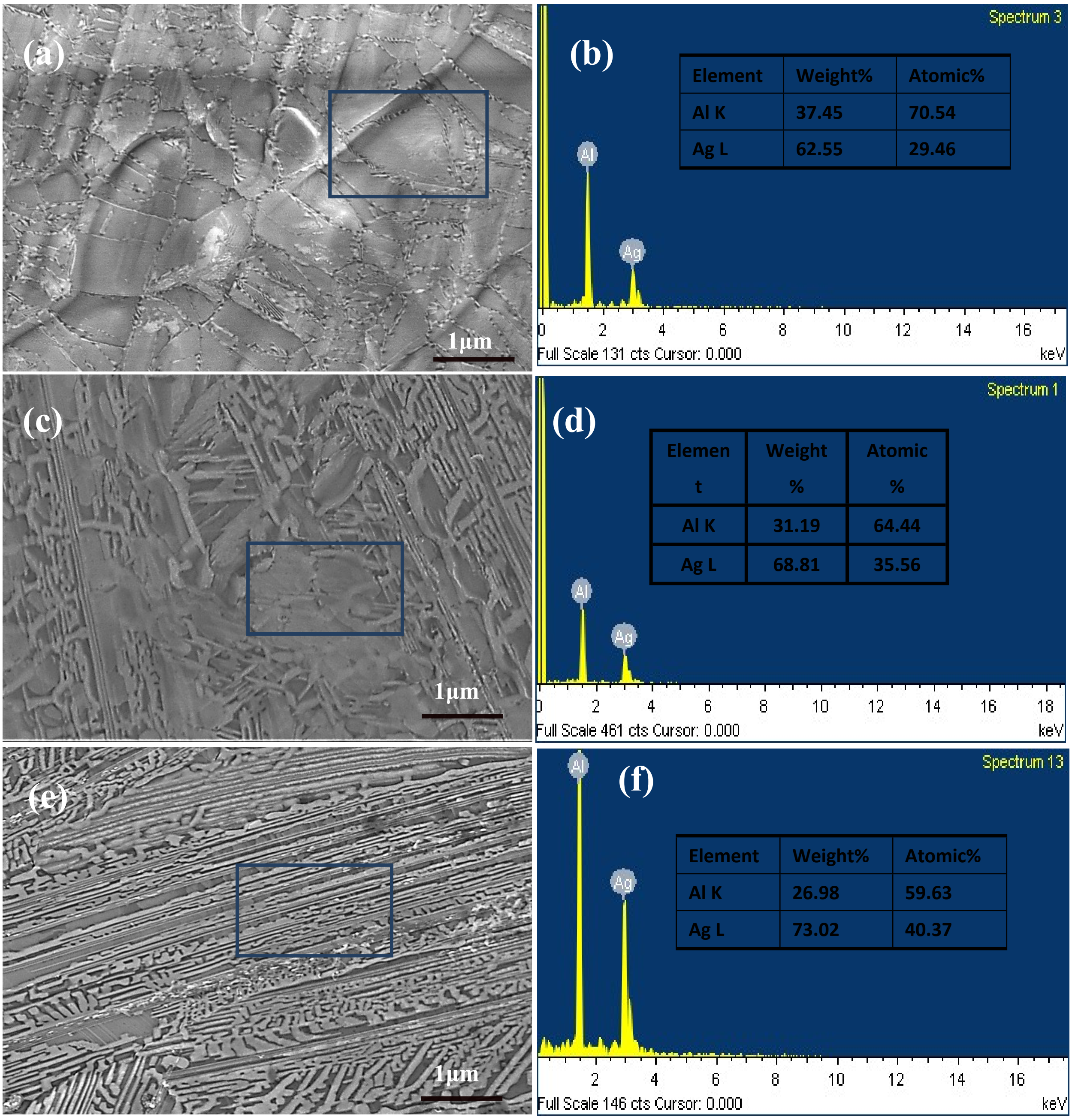
(a)
1μm
(b)
Spectrum 3
Element Weight% Atomic%
Al K 37.45 70.54
Ag L 62.55 29.46
Al
Ag
0 2 4 6 8 10 12 14 16
Full Scale 131 cts Cursor: 0.000
keV
(c)
1μm
(d)
Spectrum 1
Element Weight % Atomic %
Al K 31.19 64.44
Ag L 68.81 35.56
Al
Ag
0 2 4 6 8 10 12 14 16 18
Full Scale 461 cts Cursor: 0.000
keV
(e)
1μm
(f)
Spectrum 13
Element Weight% Atomic%
Al K 26.98 59.63
Ag L 73.02 40.37
Al
Ag
2 4 6 8 10 12 14 16
Full Scale 146 cts Cursor: 0.000
keV

**FIG. 2**

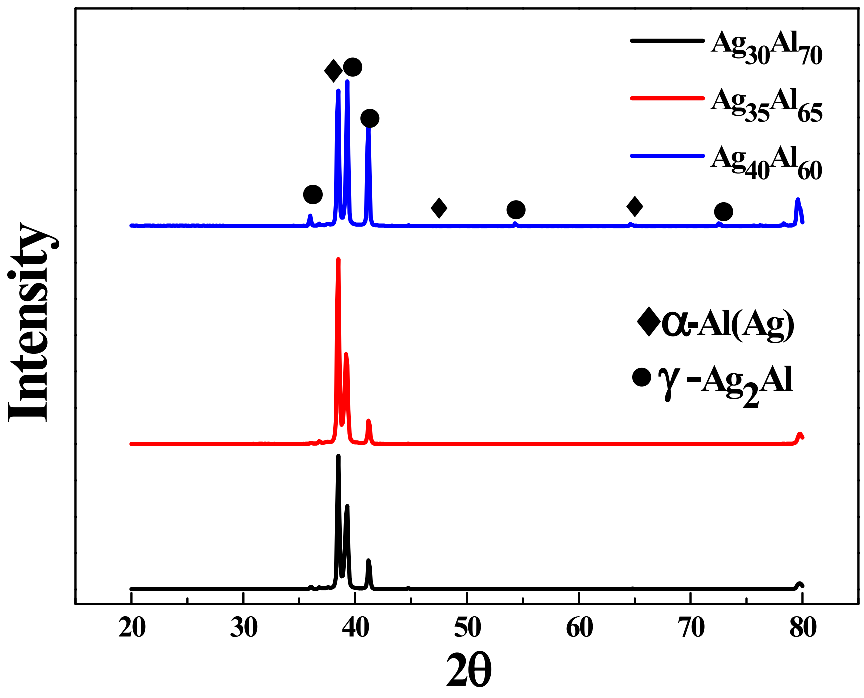
Ag30Al70
Ag35Al65
Ag40Al60
α-Al(Ag)
γ -Ag2Al
Intensity
20
30
40
50
60
70
80
2θ

FIG. 3

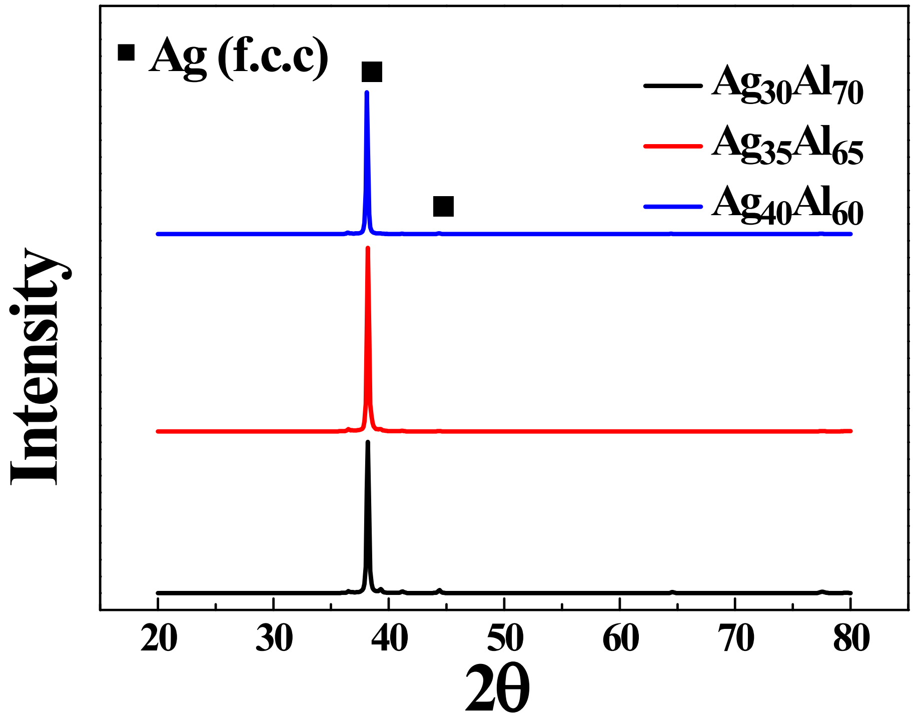

■ Ag (f.c.c)
Ag30Al70
Ag35Al65
Ag40Al60
Intensity
20
30
40
50
60
70
80
2θ


FIG. 4

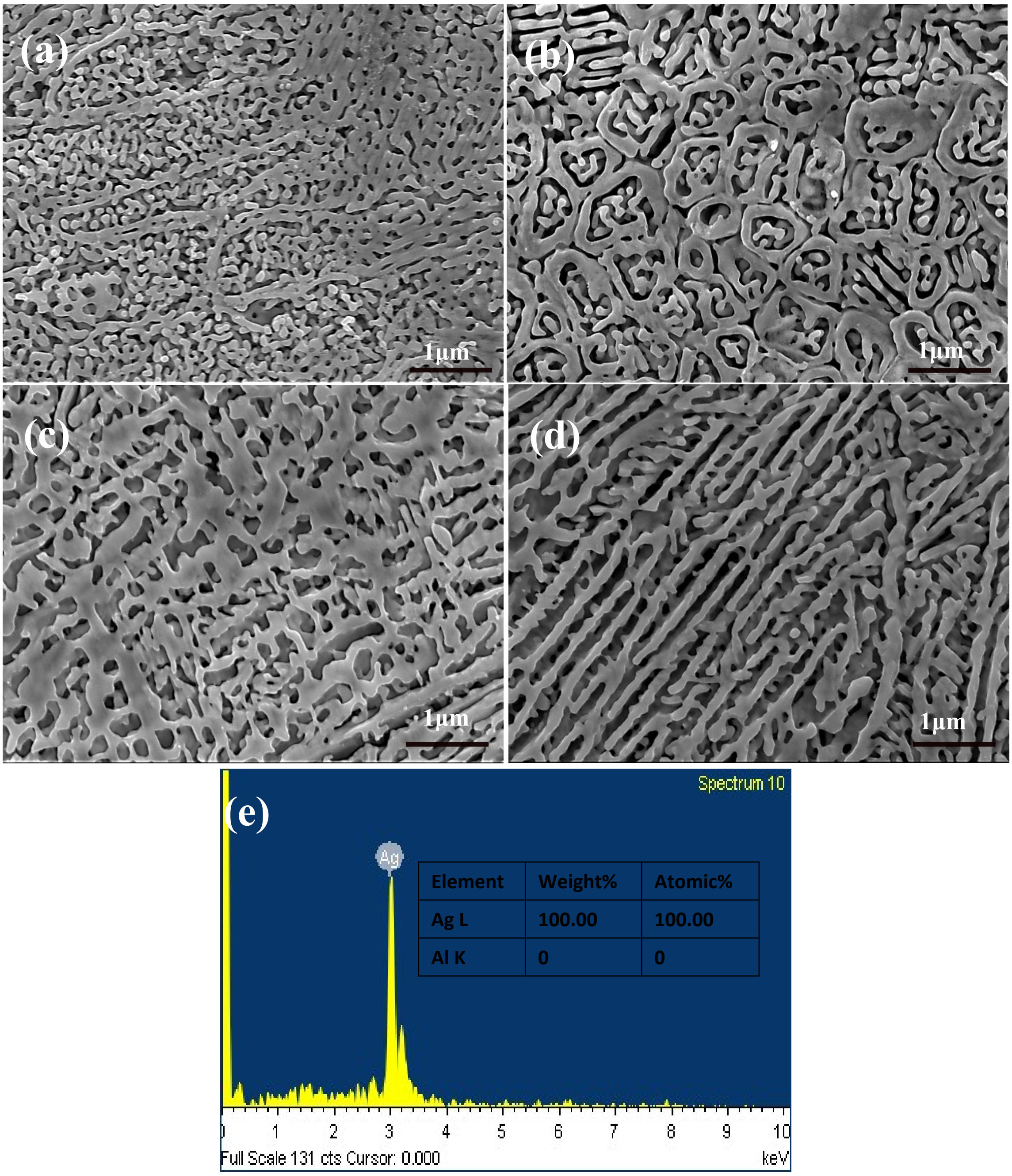
(a)
1µm
(b)
1µm
(c)
1µm
(d)
1µm
(e)
Spectrum 10
Ag
Element | Weight% | Atomic%
Ag L | 100.00 | 100.00
Al K | 0 | 0
0 1 2 3 4 5 6 7 8 9 10
Full Scale 131 cts Cursor: 0.000
keV

FIG. 5

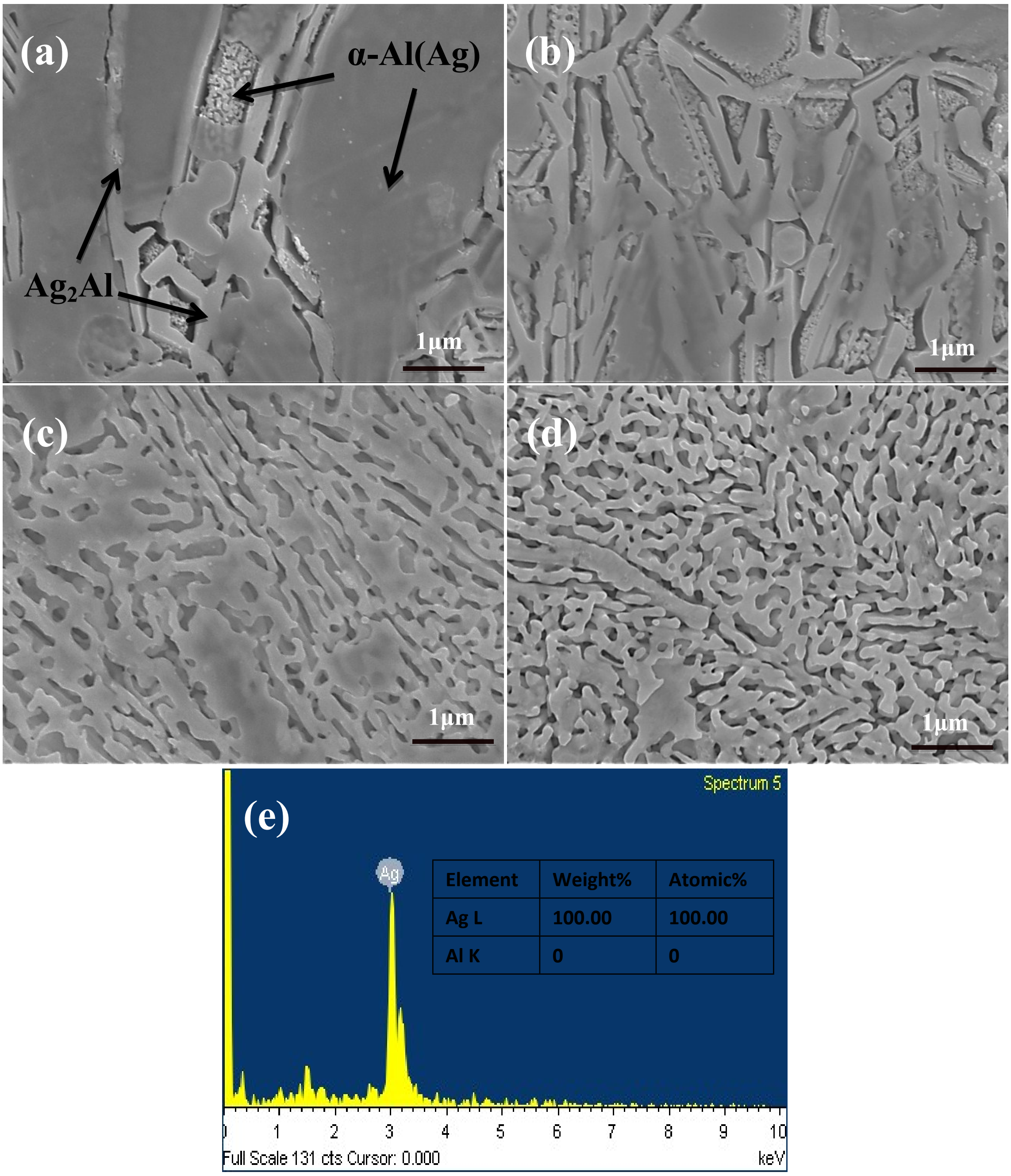
(a)
α-Al(Ag)
Ag2Al
1μm
(b)
1μm
(c)
1μm
(d)
1μm
(e)
Spectrum 5
Ag
Element
Weight%
Atomic%
Ag L
100.00
100.00
Al K
0
0
0
1
2
3
4
5
6
7
8
9
10
Full Scale 131 cts Cursor: 0.000
keV

FIG. 6

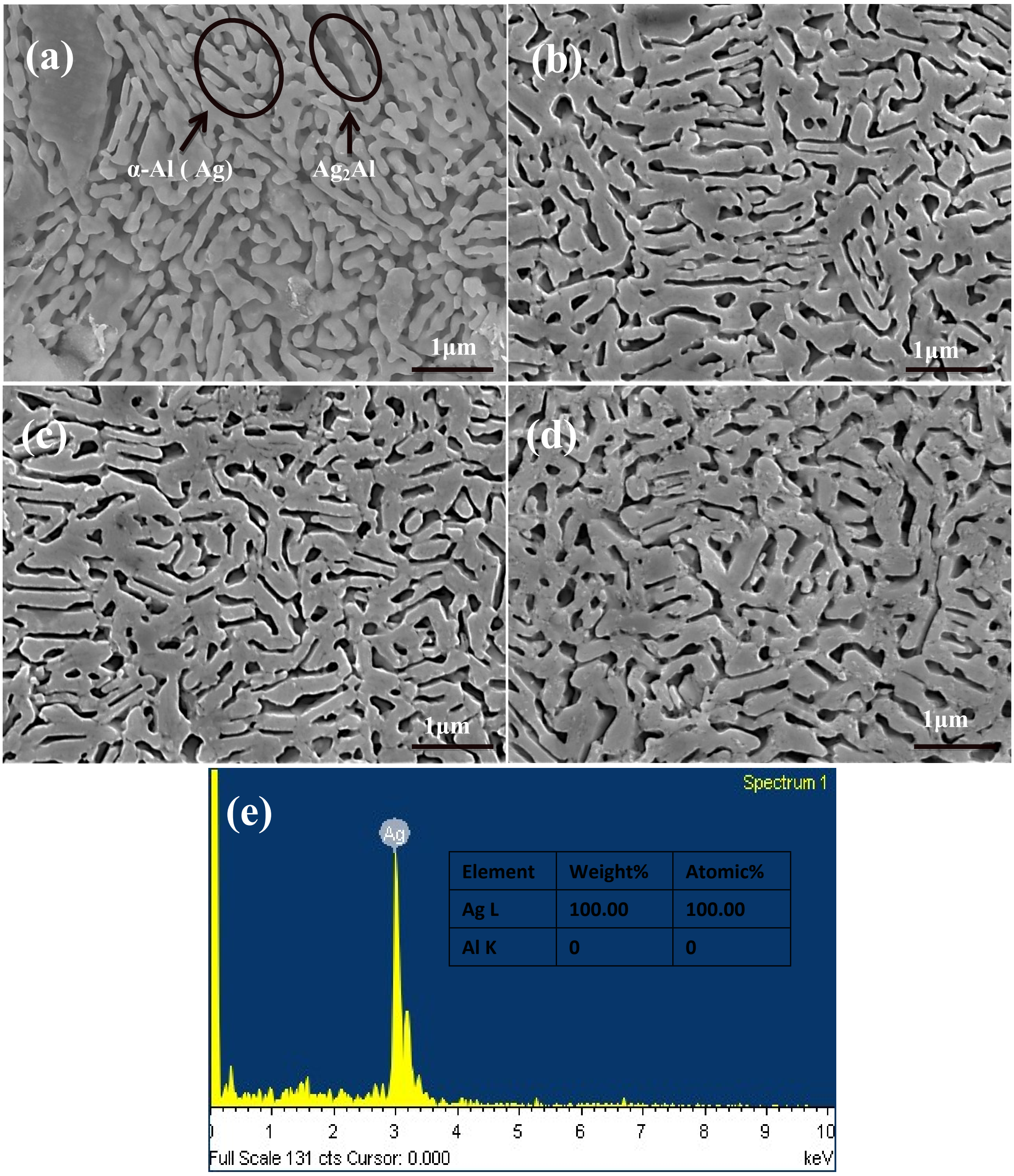
(a)
α-Al ( Ag)
Ag2Al
1μm
(b)
1μm
(c)
1μm
(d)
1μm
(e)
Spectrum 1
Ag
Element Weight% Atomic%
Ag L 100.00 100.00
Al K 0 0
1 2 3 4 5 6 7 8 9 10
Full Scale 131 cts Cursor: 0.000
keV

FIG. 7

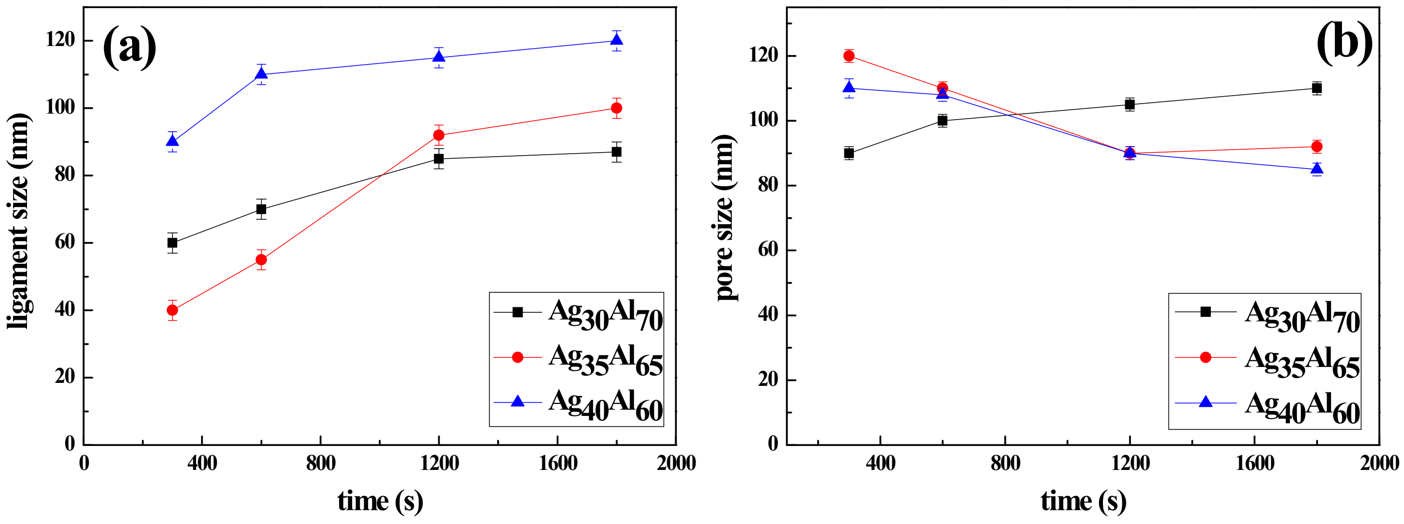
(a)
ligament size (nm)
time (s)
0
20
40
60
80
100
120
0
400
800
1200
1600
2000
$Ag_{30}Al_{70}$
$Ag_{35}Al_{65}$
$Ag_{40}Al_{60}$
(b)
pore size (nm)
time (s)
0
20
40
60
80
100
120
400
800
1200
1600
2000
$Ag_{30}Al_{70}$
$Ag_{35}Al_{65}$
$Ag_{40}Al_{60}$

**FIG. 8**

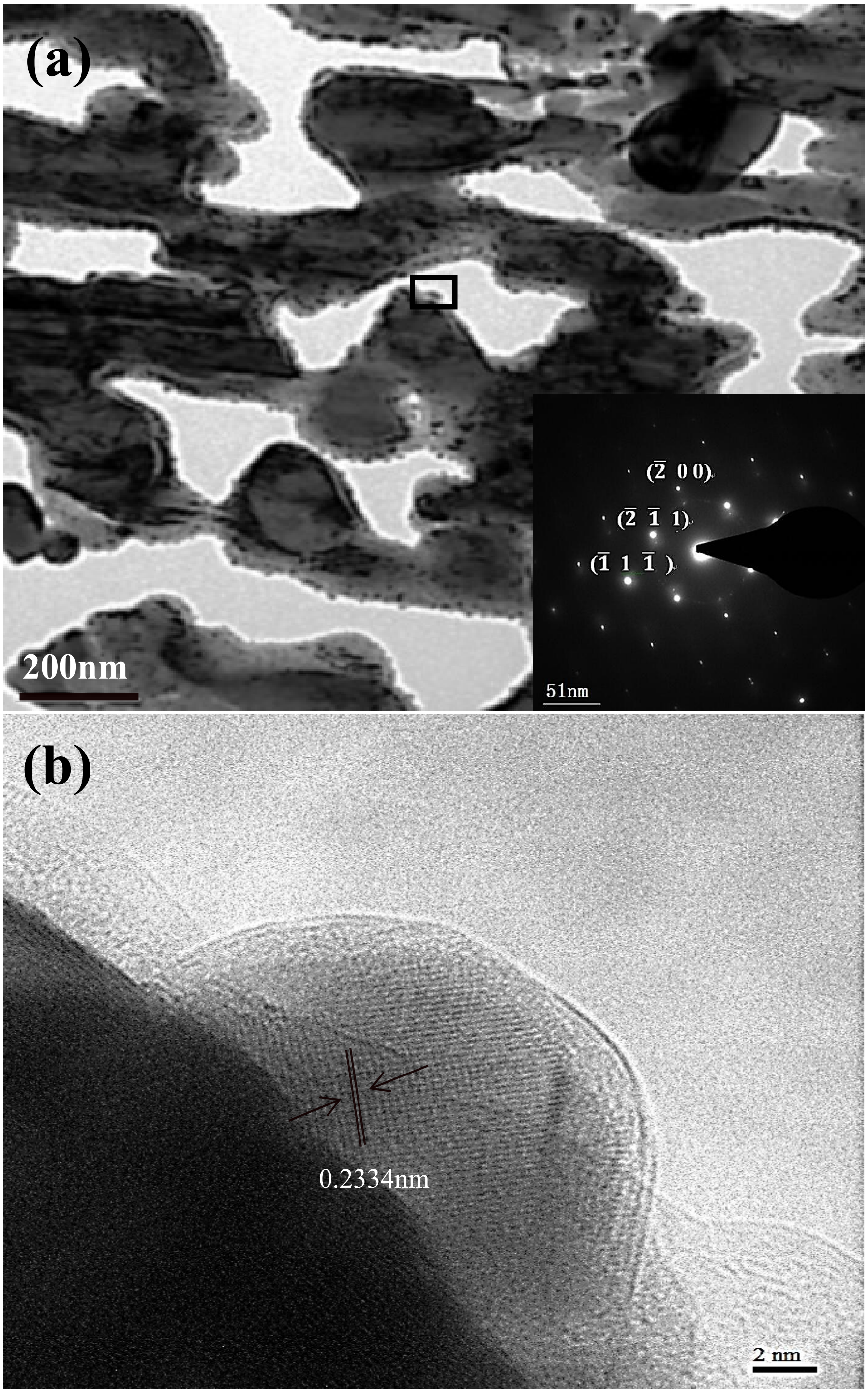


FIG. 9

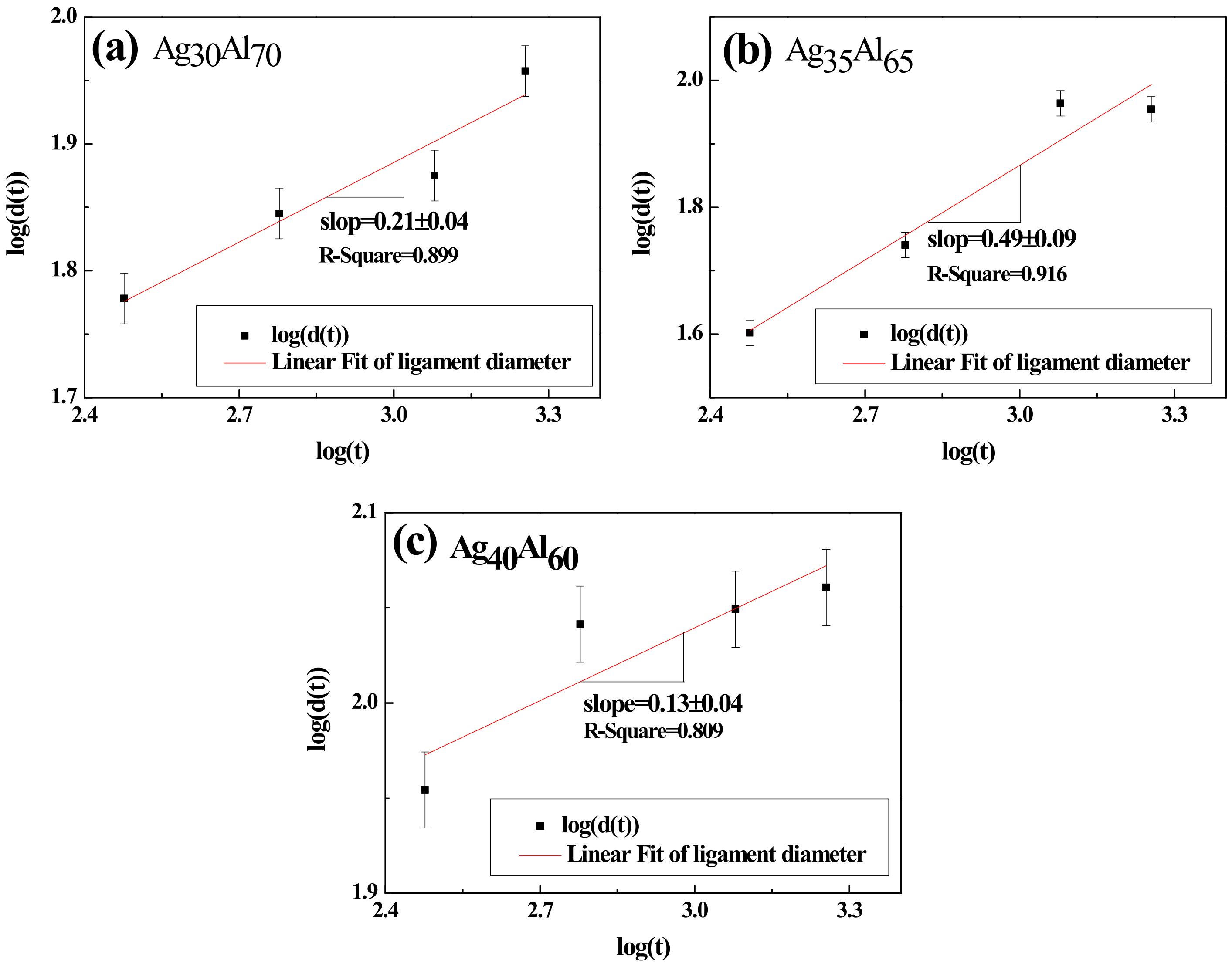

(a) Ag30Al70
slop=0.21±0.04
R-Square=0.899
log(d(t))
Linear Fit of ligament diameter
log(d(t))
log(t)
(b) Ag35Al65
slop=0.49±0.09
R-Square=0.916
(c) Ag40Al60
slope=0.13±0.04
R-Square=0.809


FIG. 10

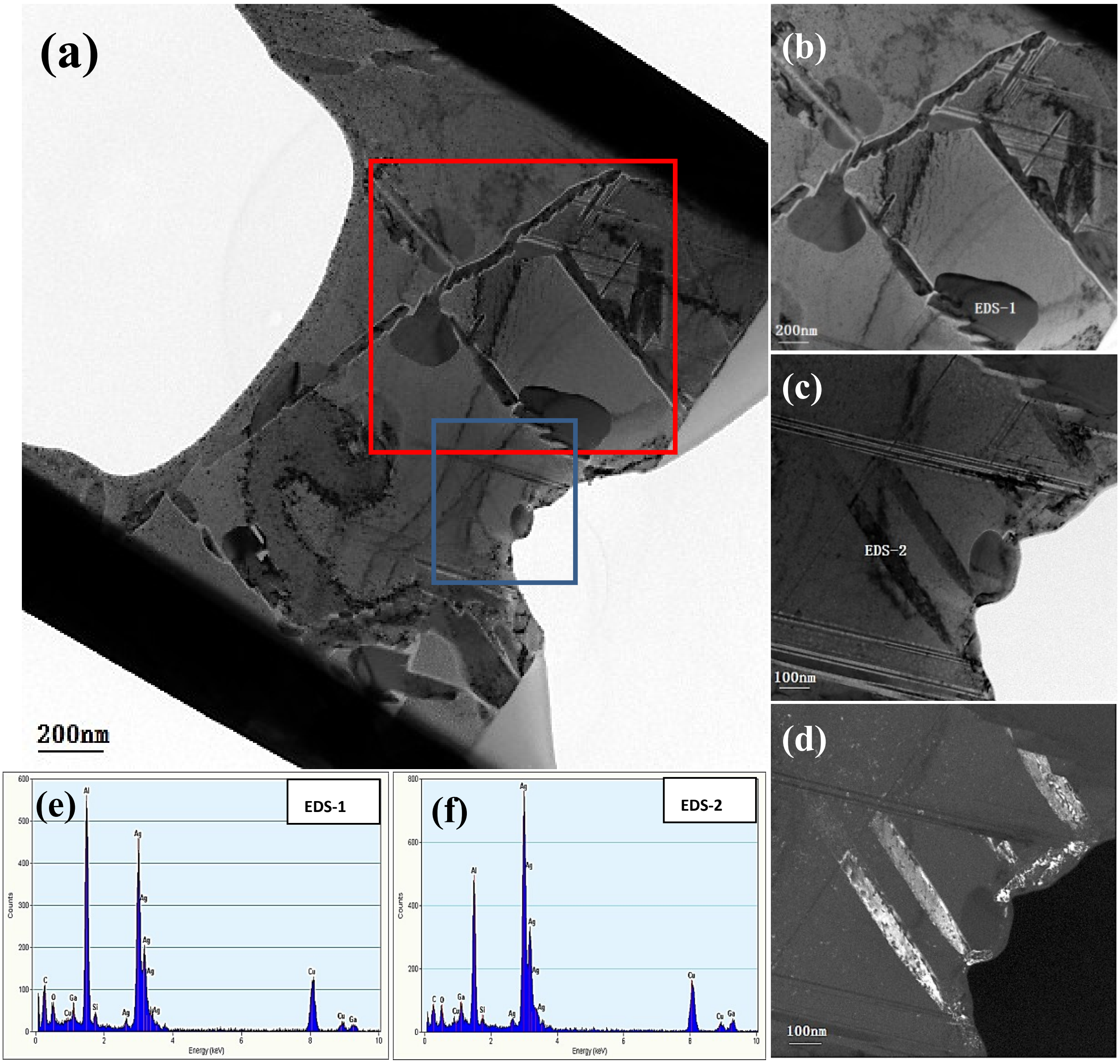
(a)
200nm
(b)
EDS-1
200nm
(c)
EDS-2
100nm
(d)
100nm
(e)
EDS-1
Energy (keV)
Counts
(f)
EDS-2
Energy (keV)
Counts

FIG. 11
